# Comparison of miRNA transcriptome of exosomes in three categories of somatic cells with derived iPSCs

**DOI:** 10.1038/s41597-023-02493-5

**Published:** 2023-09-11

**Authors:** Chunlai Yu, Mei Zhang, Yucui Xiong, Qizheng Wang, Yuanhua Wang, Shaoling Wu, Sajjad Hussain, Yan Wang, Zhizhong Zhang, Nini Rao, Sheng Zhang, Xiao Zhang

**Affiliations:** 1 University of Electronic Science and Technology of China, Chengdu, Sichuang China; 2 Binzhou Medical University, Yantai, Shandong China; 3 Guangzhou Institutes of Biomedicine and Health, Chinese Academy of Sciences, Guangzhou, Guangdong China; 4 Department of Rehabilitation Medicine, Sun Yat-sen Memorial Hospital, Sun Yat-sen University, Guangzhou, Guangdong China; 5 University of Chinese Academy of Sciences, Beijing, China; 6 GMU-GIBH Joint School of Life Sciences, Guangzhou Medical University, Guangzhou, Guangdong China

**Keywords:** miRNAs, Gene regulation, Induced pluripotent stem cells

## Abstract

Somatic cells can be reprogrammed into induced pluripotent stem cells (iPSCs) through epigenetic manipulation. While the essential role of miRNA in reprogramming and maintaining pluripotency is well studied, little is known about the functions of miRNA from exosomes in this context. To fill this research gap,we comprehensively obtained the 17 sets of cellular mRNA transcriptomic data with 3.93 × 10^10 ^bp raw reads and 18 sets of exosomal miRNA transcriptomic data with 2.83 × 10^7 ^bp raw reads from three categories of human somatic cells: peripheral blood mononuclear cells (PBMCs), skin fibroblasts(SFs) and urine cells (UCs), along with their derived iPSCs. Additionally, differentially expressed molecules of each category were identified and used to perform gene set enrichment analysis. Our study provides sets of comparative transcriptomic data of cellular mRNA and exosomal miRNA from three categories of human tissue with three individual biological controls in studies of iPSCs generation, which will contribute to a better understanding of donor cell variation in functional epigenetic regulation and differentiation bias in iPSCs.

## Background & Summary

Somatic reprogramming is a common method to manipulate cell lineage through epigenetic modification and induce somatic cells into a close embryonic stage triggered by various pluripotency master transcription factors, such as *OCT4*, *SOX2*, *KLF4*, *c-Myc*, or *NANOG*^1–4^. This somatic epigenetic manipulation has resulted in the generation of personalised induced pluripotent stem cells (iPSCs), which provide tremendous implications in regenerative medicine. During iPSCs generation, epigenetic regulation leads to differential patterns of gene expression through alterations in chromatin structure and modifications of the DNA while still sharing the same genomic sequence as its somatic cells^5–7^. Moreover, iPSCs retain epigenetic marks from their somatic source, known as “epigenetic memory”, which affects their downstream differentiation ability and inclines the differentiation to their original source^8–11^.

MiRNAs, a group of small non-coding RNAs with ~22-nt in length, were reported with solid evidence to maintain or manipulate cell lineage, which may be attributed to their ability to control factors involved in cell fate determination or epigenetic regulation^12–16^. For instance, miR-302 has been identified as a well-known gene silencer in reprogramming somatic cells into iPSCs. The miRNA function induces global DNA demethylation by repressing the expression of multiple key epigenetic regulators, such as DNMT1, MECP1/2, and HDAC2/4^12,17,18^. The miR-290 family, called embryonic stem (ES) cell-specific cell cycle regulating miRNAs, was validated to maintain the rapid proliferative state of ES cells by regulating the G1-S phase transition. Moreover, miR-9 and miR-124a, which are predominantly expressed in neurons, have been demonstrated to regulate the formation and proliferation of the neural lineage derived from ES cells based on control of STAT3 phosphorylation^19^. Interestingly, miRNAs can be regulated by various epigenetic modifications, including DNA methylation, RNA modification, and histone modifications, which further exerts extensive influence on gene expression profile^20–24^. Dysregulation of the miRNA-epigenetic feedback loop has been validated to interfere with the physiological and pathological processes, but its specific role in cell fate determination of iPSCs remains poor understood.

Exosomes, one of the smallest extracellular vesicles (EVs) secreted in various cell types, act as bioactive vesicles in cell-to-cell communication by carrying proteins, miRNAs and other factors^25,26^. In the cell microenvironment, exosomal miRNA can be taken up by neighbour cells or distant cells and subsequently regulate the epigenetics of recipient cells. It was reported that exosomal miRNAs contribute significantly to the maintenance of pluripotency or other specific cell fate in their niche^27–30^. Notably, it was unveiled that about 70% of the miRNAs identified in iPSCs were also present in iPSC- EVs^28^, which indicates miRNAs were efficiently transferred from iPSCs to EVs for regulating pluripotent signalling. While the variate of exosomal miRNAs during reprogramming was limited to investigation, their roles in regulating cell fate need to be further studied.

**Fig. 1:**
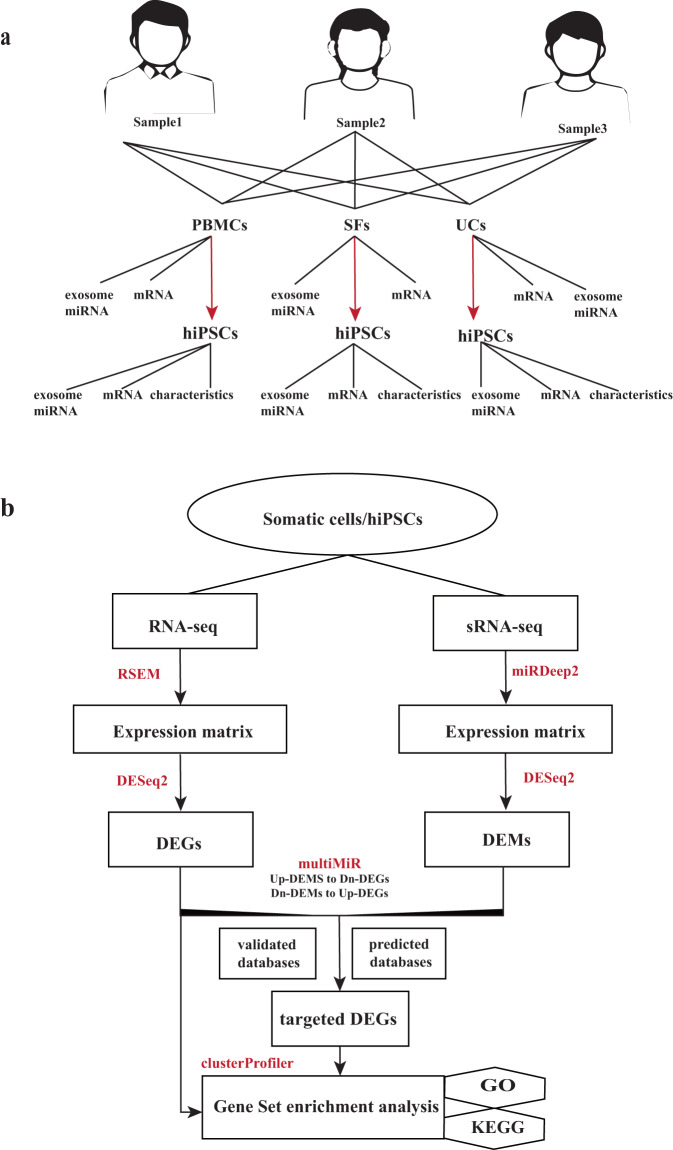
Schematic workflow of this investigation. (**a**) Exosome miRNA and total mRNA were collected from three categories of somatic cell and their derived iPSCs, along with identifying hiPSCs characteristics. (**b**) An overview of the analysis flow of miRNA and mRNA data.

To further understand the differentiation bias originating from somatic cells and the role of exosomal miRNA in regulating epigenetic heterogeneity during the generation of human iPSCs (hiPSCs), we simultaneously collected transcriptome data sets from the three most common somatic cell sources: skin fibroblasts (SFs), peripheral blood mononuclear cells (PBMCs), and urine cells (UCs), along with their derived iPSCs (Fig. 1). In order to minimise the biological variation, we recruited three healthy male donors within a similar age group (25–30 years old) and from the same genetic population (southern Han nationality represents about half of the Chinese population, approximately 10% of the world’s population^31,32^). Comparative data were generated before and after reprogramming, resulting in 17 sets of cellular RNA-Seq data and 18 sets of exosome-derived small RNA sequencing data. Subsequently, an in-house developed workflow was implemented to analyse the comparative transcriptomics data, including quality validation, differential expression analysis and gene set enrichment analysis. Our work provides a valuable resource for future investigations into donor cell variation in functional epigenetic regulation and differentiation bias in regenerative medicine.

## Methods

### Ethical approval

All samples were collected following the guidelines established by the Human Subject Research Ethics Committee at Guangzhou Institute of Biomedicine and Health (GIBH), the Chinese Academy of Sciences (CAS). The experiments were approved by the ethical committee under the approval number GIBH-IRB07-2015083. Prior to sample donation, all volunteers who donated skin, urine or blood samples had been thoroughly informed about the content, purposes, possible risks, and benefits of the experiment through a consent form, and provided their permission for genetic material data to be shared.

### Collecting and culturing the human primary somatic cells

PBMCs, SFs and UCs were isolated from three healthy males aged from 25 to 30 and satisfied specific criteria. These criteria included having a normal BMI value, no family history of genetic disease and major surgery, and not smoking and alcohol consumption. Additionally, the annual health examination reports of all three volunteers had been thoroughly reviewed, indicating their physical well-being: no chronic illnesses or infectious diseases, with the standard range for blood pressure, heart rate, standard levels for blood chemistry, including cholesterol, glucose, liver function, kidney function, and absence of any medical conditions that significantly affect bodily functions or absence of diagnosed mental disorders. Notably, all three volunteers belonged to the southern Han nationality, representing approximately half of the Chinese national population and around 10% of the global population. The cell culture conditions were referred to in the previous publications^33,34^.

### Establishment of hiPSCs from different sources

HiPSCs derived from SFs and UCs were generated based on the method described in the previous study^33^. The reprogramming procedure of UCs can be found in our previous protocol^34^. Reprogramming of human PBMCs was conducted with minor modification based on a published study^35^. Precisely, co-transfection of two episomal plasmids (pEP4-EO2SET2K and pEP4-M2L) and a vector containing hmiR302 cluster was performed in human PBMCs using Amaxa^TM^ Basic Nucleofector^TM^ Kit (Lonza), then these PBMCs were seeded on 6-well cell culture plate.

### Characterisation of the hiPSCs

The karyotypes of hiPSCs derived from three types of somatic cell sources were detected using G band techniques. The presence of inserted genes from the reprogramming plasmids were demonstrated by PCR and gel-imaging system, as described in our previous research^33^. The protocols of immunofluorescence and quantitative real-time PCR analysis were referred to our previous studies^33,34^. Approximately 1 × 10^6^ iPSCs were suspended in 100 μl Matrigel (diluted by DMEM/F12 1:1) and subcutaneously injected into the back of NOD/SCID mice. After teratoma formation, tumours were stained with haematoxylin-eosin and observed using an Olympus IX73 microscope.

### RNA extraction, library construction, and Illumina sequencing

Total RNA was isolated using TRIzol reagent (Thermo Fisher) following its standard protocols. RNA qualification, library construction and sequencing were performed as our previous publications^33,34^.

### Exosomal miRNA extraction, library construction, and Illumina sequencing

Exosomes were isolated from the cell culture medium using exoRNeasy Serum/Plasma Maxi Kit (Qiagen) following the provided instructions. HiPSCs with passage numbers ranging from 21 to 28 were used to isolate the exosomes in our experiments. Exosomal miRNA was extracted utilizing the exoRNeasy Mini Kit (Qiagen). After quantifying and qualifying the RNA, 3 μg of total RNA was used for the construction of sequencing libraries through NEBNext Multiplex Small RNA Library Prep Set for Illumina (NEB, USA). The quality of each library was assessed by the Agilent Bioanalyzer 2100 system, and sequencing was done on an Illumina Hiseq 2500/2000 platform.

### Preprocessing of RNA sequencing data

Raw sequencing data were processed by fastp v0.20.1^36^ with default parameters to remove adapter sequences, low-quality reads and short-length sequences. The resulting clean reads were then mapped to the human genome hg38 to quantify global gene expression using the expectation-Maximization method implemented in RSEM v1.2.22. Gene count and transcripts per million (TPM) matrix information was obtained for each sample. After filtering low-expression genes with an average expression less than 1, the log_2_(CPM + 1) values of each sample were used for the principal component analysis (PCA) and the correlation coefficient calculation. Differentially expressed genes (DEGs) between somatic cells and their derived hiPSCs were determined by DESeq 2 v1.30.1^37^. DEGs were identified with an adjusted P-value < 0.05 and the absolute value of log_2_FoldChange > 1. While TPM matrix information was used to evaluate the expression levels of differentiation and pluripotency related genes. Gene set enrichment analysis was conducted by clusterProfiler v4.4.4^38^ based on Gene Ontology (GO) and the Kyoto Encyclopedia of Genes and Genomes (KEGG) databases.

### Preprocessing of small RNA sequencing data

FastQC (http://www.bioinformatics.babraham.ac.uk/projects/fastqc/) was utilized to verify the sequence quality by assessing parameters such as Q20, Q30, GC-content and adapter sequences. The raw data were mapped to the human genome hg38 and human miRNA sequences from miRBase^39^ v22 to predict novel miRNAs and evaluate the expression levels of known miRNAs by miRDeep^40^ v2.0.1.2 in multiple samples mode. The mapping number of each read in the human genome hg38 was constrained by a maximum of 5. Low-expression miRNAs with an average expression of less than 1 were filtered out, and then log_2_(CPM + 1) values of miRNA in each sample were used to perform the principal component analysis (PCA) and calculate the correlation coefficient. Differentially expressed miRNAs (DEMs) were identified using the same method as for DEGs. The miRNA targeted genes were analysed by multiMiR^41^ v1.12.0 based on its database v2.3, which incorporated three validated miRNA-target interactions databases (miRecoord, miRtarBase and TarBase) and 8 predicted miRNA-target interactions databases (DIANA-microT-CDS, RIMMo, MicroCosm, miRDB, PicTar, PITA and targetScan). The down-regulated DEGs were used to explore the up-regulated DEMs target genes, while the up-regulated DEGs were used to explore the down-regulated DEMs target genes. The targeted genes found in validated databases or in more than three predicted databases underwent GO and KEGG pathway enrichment, as described above.

## Data Records

Our raw data, consisting of 17 RNA-seq and 18 exosomes’ small RNA-seq data sets, was stored in the Genome Sequence Archive^42^ in National Genomics Data Center (NGDC)^43^ with the accession number HRA003697^44^. The corresponding expression matrix information was deposited in NGDC of China National Center for Bioinformation with the accession number PRJCA013662^45^.

## Technical Validation

### The characteristics of hiPSCs

**Fig. 2:**
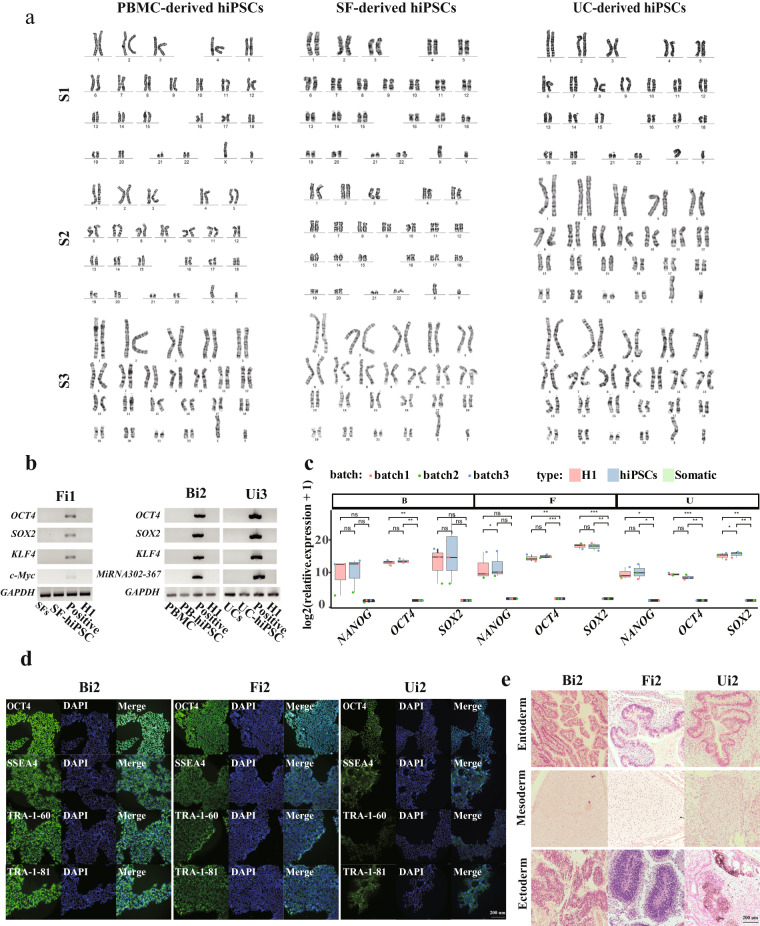
Characteristics of hiPSCs. (**a**) The karyotype of hiPSCs derived from three somatic cell types with three samples. (**b**) The presence of exogenous episomal DNA in hiPSC was identified by agarose gel electrophoresis. Somatic cells transfected by episomal DNA served as the positive control, while the H1 cell line and somatic cells served as the negative controls; GAPDH served as the internal reference. B: PBMCs, F: SFs, U: UCs, i: hiPSCs. The specimens Fi1, Bi2 and Ui3 were chosen to show the results. (**c**) The expression levels of *NANOG* *OCT4*, and *SOX2* in somatic cells, their derived hiPSCs and H1 cells were evaluated by qRT-PCR. The gene expression level in each sample was detected triple times, and its mean expression was used as its expression value. Each point represents a sample value. The P-value was calculated by Student’s t-test. *P < 0.05, **P < 0.01, ***P < 0.001. (**d**) The detection of OCT4, SOX2, SSEA4, TRA-1-60 and TRA-1-81 by immunostaining. scale bar: 200 um. (**e**) The histology of teratomas induced from hiPSCs derived from different somatic cells. The hiPSCs generated from sample 2 were chosen to show as an example. Teratomas were stained with H&E.

Karyotype analysis indicated that the chromosome profiles of hiPSCs derived from PBMCs, SFs and UCs were without abnormality (Fig. 2a). The exogenous episomal DNA (*OCT4*, *SOX2*, *KLF4*, *MicroRNA302-367* or *c-Myc*) was absent in all three somatic cell-derived hiPSCs (Fig. 2b). Compared with their respective somatic cells, the mRNA expression levels of *OCT4*, *SOX2* and *NANOG* were significantly increased in hiPSCs from three categories (Fig. 2c). Immunofluorescence result revealed higher expression levels of the pluripotent protein markers (OCT4, SSEA4, TRA-1-60, and TRA-1-81) in hiPSCs derived from the three somatic cell types compared to their respective somatic cells (Fig. 2d). The pluripotent potential of hiPSCs derived from the three types of somatic cells was further investigated by teratoma formation, which exhibited their ability to differentiate into three germ-layer (Fig. 2e). These results illustrated that our hiPSCs have similar pluripotent characteristics to human embryonic stem cells.

### Quality control of RNA sequencing data

High-throughput RNA sequencing generated 1.2~2.0 × 10^7^ raw reads per sample, with the Q20 > 0.95, Q30 > 0.90, GC-content close to 0.50, and the mean length of 149 bp for clean reads(Table 1). Most of the pair reads were aligned to the hg38 genome, with the sample align rate ranging from 66% to 88% and the unique mapping rate ranging from 15% to 22%, much lower than that of the multiple mapping rate (Table 2).

**Table 1:** RNA-seq data quality summary.

sampleid	before_filtering					after_filtering						
	total_reads	total_bases	q20_rate	q30_rate	GC_content	total_reads	total_bases	q20_rate	q30_rate	read1_mean length	read2_mean length	GC_content
Bi1	167,085,244	25,062,786,600	0.97	0.92	0.5	165,215,442	24,690,424,461	0.97	0.92	149	149	0.5
Bi2	198,213,630	29,732,044,500	0.98	0.95	0.51	197,135,992	29,346,669,242	0.98	0.95	148	148	0.51
Bi3	154,626,132	23,193,919,800	0.98	0.95	0.5	153,744,964	22,943,020,341	0.98	0.95	149	149	0.5
Bs1	158,272,448	23,740,867,200	0.97	0.92	0.51	156,522,756	23,370,081,320	0.97	0.92	149	149	0.51
Bs2	125,392,896	18,808,934,400	0.97	0.91	0.51	124,162,240	18,543,921,985	0.97	0.92	149	149	0.51
Bs3	148,912,774	22,336,916,100	0.98	0.95	0.5	148,124,384	22,093,629,684	0.98	0.95	149	149	0.5
Fi1	147,477,168	22,121,575,200	0.97	0.92	0.5	145,817,296	21,774,630,298	0.97	0.92	149	149	0.5
Fi2	148,209,424	22,231,413,600	0.97	0.92	0.5	146,779,778	21,894,281,882	0.97	0.92	149	149	0.5
Fs1	167,903,624	25,185,543,600	0.97	0.92	0.51	166,251,896	24,812,269,267	0.97	0.93	149	149	0.51
Fs2	119,458,608	17,918,791,200	0.97	0.91	0.52	118,244,480	17,658,677,928	0.97	0.92	149	149	0.52
Fs3	136,870,536	20,530,580,400	0.96	0.91	0.52	135,223,114	20,174,078,894	0.97	0.91	149	149	0.52
Ui1	142,213,316	21,331,997,400	0.97	0.92	0.5	140,645,662	20,993,560,343	0.97	0.92	149	149	0.5
Ui2	182,961,012	27,444,151,800	0.98	0.95	0.5	181,910,786	27,106,815,710	0.98	0.95	149	149	0.5
Ui3	167,475,322	25,121,298,300	0.98	0.95	0.5	166,480,576	24,826,805,145	0.98	0.95	149	149	0.5
Us1	157,850,182	23,677,527,300	0.97	0.92	0.5	155,991,542	23,306,041,752	0.97	0.93	149	149	0.5
Us2	145,830,832	21,874,624,800	0.97	0.91	0.51	144,310,566	21,554,165,472	0.97	0.92	149	149	0.51
Us3	150,623,110	22,593,466,500	0.96	0.9	0.49	148,564,170	22,139,867,523	0.97	0.91	149	149	0.49

Q20/30 means the average quality value of nucleotide in reads above 20/30. B: PBMCs, F: skin fibroblasts, U: urine cells, i: iPSCs, s: somatic cells, 1: sample1, 2: sample2, 3: sample3.

**Table 2:** Summary of RNA-seq reads mapping results.

sampleid	paired_reads	failed	uniq_align	mul_align	%failed	%uniq_align	%mul_align	%overall_align
Bs1	78,261,378	15,565,359	13,876,533	48,819,486	19.89	17.73	62.38	80.11
Fs1	83,125,948	15,145,147	16,124,951	51,855,850	18.22	19.40	62.38	81.78
Us1	77,995,771	15,521,232	14,698,685	47,775,854	19.90	18.85	61.25	80.10
Bi1	82,607,721	18,152,362	15,432,257	49,023,102	21.97	18.68	59.34	78.03
Fi1	72,908,648	16,418,354	13,467,377	43,022,917	22.52	18.47	59.01	77.48
Ui1	70,322,831	14,608,891	12,916,551	42,797,389	20.77	18.37	60.86	79.23
Bi3	76,872,482	17,838,929	14,025,668	45,007,885	23.21	18.25	58.55	76.79
Bs3	74,062,192	15,404,209	13,775,641	44,882,342	20.08	18.60	60.60	79.20
Us3	74,282,085	24,942,273	11,654,384	37,685,428	33.58	15.69	50.73	66.42
Ui3	83,240,288	18,751,705	15,045,643	49,442,940	22.53	18.07	59.40	77.47
Fs3	67,611,557	8,837,005	14,370,893	44,403,659	13.07	21.26	65.67	86.93
Bs2	62,081,120	13,230,579	11,020,800	37,829,741	21.31	17.75	60.94	78.69
Bi2	98,567,996	24,434,449	17,722,144	56,411,403	24.79	17.98	57.23	75.21
Fi2	73,389,889	15,725,880	13,506,663	44,157,346	21.43	18.40	60.17	78.57
Us2	72,155,283	14,419,793	13,421,532	44,313,958	19.98	18.60	61.41	80.02
Ui2	90,955,393	21,742,248	16,399,825	52,813,320	23.90	18.03	58.07	76.10
Fs2	59,122,240	7,432,055	12,316,937	39,373,248	12.57	20.83	66.60	87.43

uniq_align: unique alignment, mul_align: multiple alignment, overall_align: overall alignment.

### Genes expression analysis

**Fig. 3:**
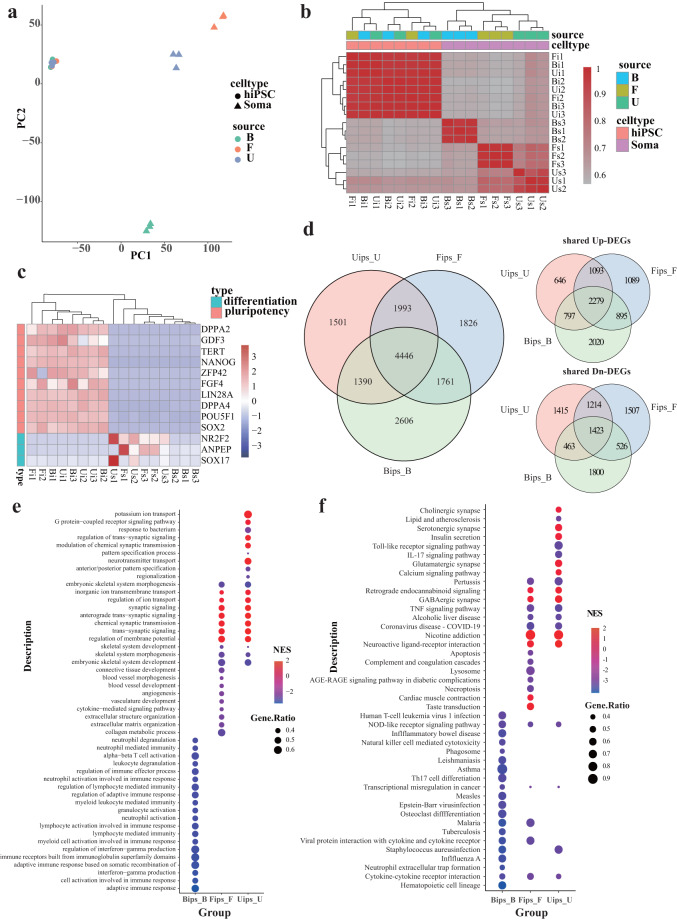
Gene expression among somatic cells and hiPSCs. (**a**) Principal components analysis of different somatic cells and their derived hiPSCs. (**b**) Correltion analysis of different somatic cells and their derived hiPSCs. (**c**) The mRNA expression levels of the pluripotency and differentiation related genes. (**d**) Venn plot of DEGs among the three groups. Bips_B: PBMCs-derived hiPSCs Vs. PBMCs, Fips_F: SFs-derived hiPSCs Vs. SFs, Uips_U: UCs-derived hiPSCs Vs. UCs. (**e,f**) Gene set enrichment analysis of DEGs based on biological process GO and KEGG databases, respectively. The top 20 enriched pathways of each group were displayed.

Gene expression analysis was performed using three biologically replicated samples, except that SFs-derived hiPSCs had only two samples. The correlation coefficients calculated based on Pearson correlation and PCA analysis implied good repeatability within the biological replicates and high similarity among hiPSCs derived from the three kinds of somatic cells (Fig. 3a,b). The gene expression levels of the pluripotency related genes (*PPA2*, *GDF3*, *TERT*, *NANOG*, *ZFP42*, *FGF4*, *LIN28A*, *DPPA4*, *POU5F1*, and *SOX2*) were significantly higher in hiPSCs than that in somatic cells, whereas the gene expression levels of differentiation related genes (*NR2F2*, *ANPEP*, and *SOX17)* were significantly lower (Fig. 3c). The analysis of differentially expressed genes (DEGs) between somatic cells and their derived hiPSCs indicated significant changes from a differentiated state to a pluripotency state. In these sets, a total of 10,203 genes, 10,026 genes and 9,330 genes were identified as DEGs (Table 3), respectively, including 4446 shared DEGs with 2279 consistently up-regulated DEGs and 1423 consistently down-regulated DEGs. (Fig. 3d). In addition, the top 20 pathways identified in the enrichment analysis of DEGs based on the biological process GO and KEGG indicated that immune-related pathways were mostly down-regulated in PBMCs-derived hiPSCs compared to PBMCs. Up-regulated synaptic signalling and down-regulated embryonic skeletal system development were both observed in the SFs and UCs categories (Fig. 3e,f).

**Table 3:** The number of differently expressed genes (DEGs) between hiPSCs and somatic cells.

hiPSCs	somactic cells	up_DEGs	down_DEGs	sum
BiPS	PBMCs	5991	4212	10203
FiPS	SFs	5356	4670	10026
UiPS	UCs	4815	4515	9330

BiPS: PBMCs-derived hiPSCs, FiPS:SFs-derived hiPSCs, UiPS: UCs-derived hiPSCs. The genes with |log2FoldChange (FC)| ≥ 1, and an adjusted P-value < 0.05 were determined as DEGs.

### Processing the small RNA sequencing data

**Fig. 4:**
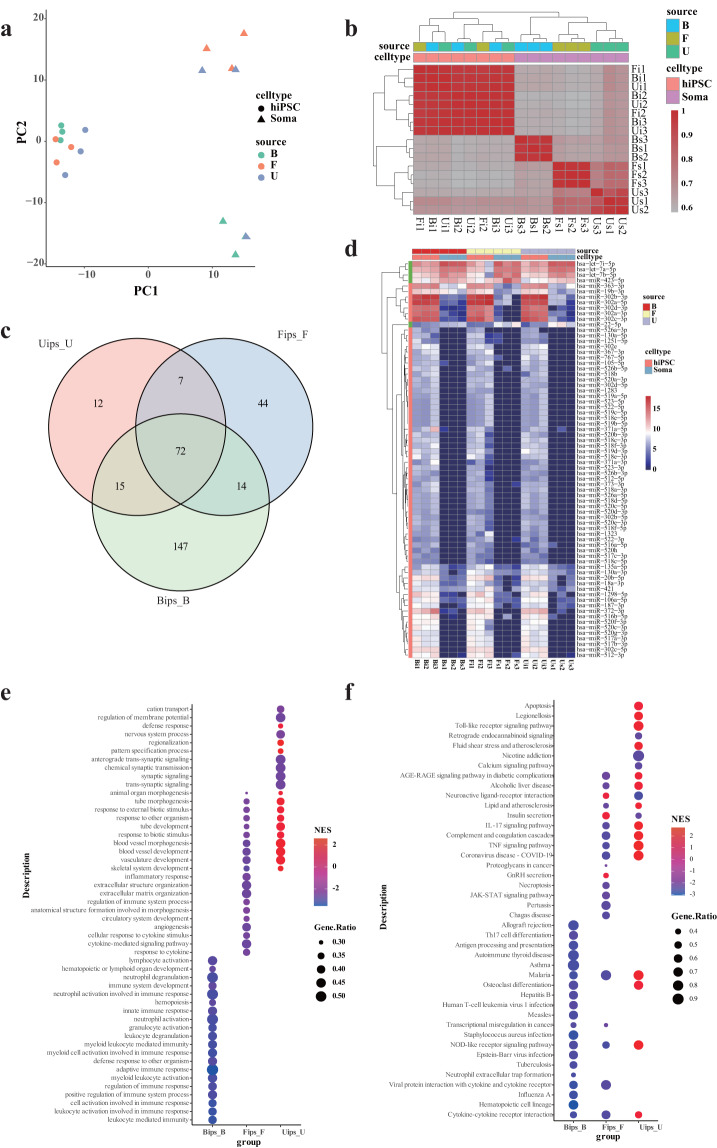
Expression of miRNA in exosomes from somatic cells and hiPSCs. (**a**) Principal components analysis applied to different somatic cells and their derived hiPSCs. (**b**) Correlation analysis of different somatic cells and their derived hiPSCs. (**c**) Venn plot of differentially expressed miRNA (DEMs) among the three groups, Bips_B: PBMCs-derived hiPSCs Vs. PBMCs, Fips_F: SFs-derived hiPSCs Vs. SFs, Uips_U: UCs-derived hiPSCs Vs. UCs. (**d**) The expression profile of the shared DEMs (**e,f**) Gene set enrichment analysis of DEGs targeted by DEMs based on GO and KEGG databases, respectively. The top 20 enriched pathways of each group were shown.

A total of 2.83 × 10^7^ raw reads were generated, ranging from 1.1 to 2.2 × 10^6^ raw reads per sample. The Q20 rate was more than 0.99, the Q30 rate was more than 0.98, and the GC content of raw reads with a mean length of 50 bp was approximately 0.53 (Table 4). In summary, the small RNA-seq sequencing data have quite good quality. The mapping rate of small RNA reads to the hg38 genome per sample ranged from 19% to 62%, while the mapping rate of reads to human miRNA sequences ranged from 0.5% to 12%, with an average rate of 2.50% (Table 5). According to data GSE216556^46^ deposited on the NCBI GEO database, the mapping rates of small RNA sequences extracted from exosomes to human miRNA sequences ranged from 1.26% to 2.68%, indicating a lower alignment rate of miRNA from exosomes compared to the cellular datasets. The results of correlation coefficients and the PCA analysis implied good repeatability within the biological replicates, except for sample Us3, which displays a closer relationship to the PBMCs exosomes (Fig. 4a,b). The exosomal miRNA data generated from three categories of hiPSCs exhibited a high degree of similarity, vastly different from their somatic miRNA samples. A total of 248, 137 and 106 miRNAs were separately identified as differentially expressed miRNAs (DEMs) in these three groups, including shared 72 DEMs with 68 consistently up-regulated DEMs and 5 consistently down-regulated DEMs (Table 6, Figs. 3d, 4c). The items of DEGs targeted by DEMs were summarised in Table 7. The gene set enrichment analysis of targeted DEGs for each group based on biological process GO, and KEGG was conducted and revealed similar enriched pathways to DEGs (Table 8). The top 20 enriched pathways were shown in Fig. 4e,f.

**Table 4:** Quality summary of small RNA-seq data.

Sampleid	total_reads	total_bases	q20_bases	q30_bases	Q20_rate	Q30_rate	mean_length	gc_content
Bi1	15,983,996	799,199,800	796,826,629	792,870,660	0.997	0.992	50	0.53
Bi2	18,872,729	943,636,450	938,077,306	929,769,268	0.994	0.985	50	0.53
Bi3	15,732,231	786,611,550	785,093,177	781,918,272	0.998	0.994	50	0.54
Bs1	21,176,010	1,058,800,500	1,055,404,393	1,049,312,021	0.997	0.991	50	0.53
Bs2	21,911,464	1,095,573,200	1,092,947,383	1,088,359,267	0.998	0.993	50	0.54
Bs3	11,545,471	577,273,550	574,355,218	568,894,695	0.995	0.985	50	0.52
Fi1	14,233,184	711,659,200	709,793,867	706,682,059	0.997	0.993	50	0.52
Fi2	14,938,057	746,902,850	745,086,133	742,084,016	0.998	0.994	50	0.53
Fi3	11,886,246	594,312,300	592,697,475	589,236,954	0.997	0.991	50	0.54
Fs1	13,429,220	671,461,000	670,324,681	668,298,334	0.998	0.995	50	0.54
Fs2	19,247,873	962,393,650	959,346,447	954,100,077	0.997	0.991	50	0.54
Fs3	12,756,575	637,828,750	636,326,501	633,069,197	0.998	0.993	50	0.54
Ui1	16,088,130	804,406,500	802,295,973	798,437,967	0.997	0.993	50	0.53
Ui2	14,219,139	710,956,950	707,262,402	700,814,988	0.995	0.986	50	0.54
Ui3	12,721,163	636,058,150	632,932,608	627,133,372	0.995	0.986	50	0.53
Us1	14,992,844	749,642,200	747,796,975	744,619,620	0.998	0.993	50	0.53
Us2	21,346,203	1,067,310,150	1,063,346,899	1,056,552,796	0.996	0.99	50	0.53
Us3	11,545,471	577,273,550	574,355,218	568,894,695	0.995	0.985	50	0.52

**Table 5:** Summary of small RNA-seq reads mapping results.

sampleid	mapping to genome					mapping to human miRNA				
	total	mapped	unmapped	%mapped	%unmapped	total	mapped	unmapped	%mapped	%unmapped
total	271,360,581	84,789,895	186,570,686	31.246	68.754	269,729,573	6,754,549	262,975,024	2.504	97.496
Bi1	15,762,375	3,908,311	11,854,064	24.795	75.205	15,685,441	139,053	15,546,388	0.887	99.113
Bi2	18,638,556	6,312,046	12,326,510	33.866	66.134	18,490,952	278,626	18,212,326	1.507	98.493
Bi3	15,416,194	3,949,971	11,466,223	25.622	74.378	15,388,520	116,991	15,271,529	0.760	99.240
Bs1	20,929,474	6,005,381	14,924,093	28.693	71.307	20,838,848	539,053	20,299,795	2.587	97.413
Bs2	21,300,568	12,536,808	8,763,760	58.857	41.143	20,928,490	1,003,553	19,924,937	4.795	95.205
Bs3	10,731,725	5,108,306	5,623,419	47.600	52.400	10,715,614	957,401	9,758,213	8.935	91.065
Fi1	14,093,922	3,743,562	10,350,360	26.562	73.438	14,021,907	128,966	13,892,941	0.920	99.080
Fi2	14,685,735	3,276,150	11,409,585	22.308	77.692	14,446,718	175,314	14,271,404	1.214	98.786
Fi3	8,118,568	5,057,441	3,061,127	62.295	37.705	8,107,302	934,318	7,172,984	11.524	88.476
Fs1	12,826,098	3,468,919	9,357,179	27.046	72.954	12,772,459	186,381	12,586,078	1.459	98.541
Fs2	18,768,802	2,814,758	15,954,044	14.997	85.003	18,645,871	77,853	18,568,018	0.418	99.582
Fs3	12,552,154	3,924,875	8,627,279	31.269	68.731	12,527,848	523,224	12,004,624	4.176	95.824
Ui1	15,995,141	4,334,943	11,660,198	27.102	72.898	15,931,942	131,848	15,800,094	0.828	99.172
Ui2	13,973,256	4,611,054	9,362,202	32.999	67.001	13,931,071	213,275	13,717,796	1.531	98.469
Ui3	12,142,115	3,730,789	8,411,326	30.726	69.274	12,124,356	179,931	11,944,425	1.484	98.516
Us1	14,557,860	2,909,650	11,648,210	19.987	80.013	14,479,603	82,197	14,397,406	0.568	99.432
Us2	20,136,313	3,988,625	16,147,688	19.808	80.192	19,977,017	129,164	19,847,853	0.647	99.353
Us3	10,731,725	5,108,306	5,623,419	47.600	52.400	10,715,614	957,401	9,758,213	8.935	91.065

**Table 6:** The number of DEMs between hiPSCs and somatic cells.

hiPSCs	somatic cells	up_DEMs	down_DEMS	sum
BiPS exosomes	PBMCs exosomes	169	79	248
FiPS exosomes	SFs exosomes	109	28	137
UiPS exosomes	UCs exosomes	79	27	106

DEMs: differently expressed miRNAs, BiPS: PBMCs-derived hiPSCs, FiPS:SFs-derived hiPSCs, UiPS: UCs-derived hiPSCs. The miRNAs with |log2 fold change (FC)| ≥ 1, and an adjusted P-value < 0.05 were determined as DEMs.

**Table 7:** The number of DEGs targeted by DEMs.

Type	Database number	B_dnDEM2upDEG	B_upDEM2dnDEG	F_dnDEM2upDEG	F_upDEM2dnDEG	U_dnDEM2upDEG	U_upDEM2dnDEG
validated miRNA-gene interaction	>0	18,902	15,689	6,562	16,317	9,648	8,318
	>1	1980	1,207	581	1,561	1,041	917
	>2	38	77	14	41	23	55
predicted miRNA-gene interaction	>3	2,353	3,535	523	3,280	1,027	2,354
	>4	2,353	3,535	523	3,280	1,027	2,354
	>5	251	270	82	346	164	300
	>6	31	20	6	38	17	35

B: PBMCs-derived hiPSCs vs. PBMCs, F: SFs-derived hiPSCs vs. SFs, U: UCs-derived hiPSCs vs. UCs. dnDEM2upDEG illustrated up-regulated DEGs are used to search the targeted genes of down-regulated DEMs, upDEM2dnDEG illustrated down-regulated DEGs are used to search the targeted genes of up-regulated DEMs.

**Table 8:** The number of enriched pathways of DEGs and their targeting DEGs based on biological process GO and KEGG.

Database	Type	Bips_B	Fips_F	Uips_U
GO:BP	DEGs	1,175	523	668
	DEMs2DEGs	1,057	674	668
	Shared	969	386	474
KEGG	DEGs	98	60	74
	DEMs2DEGs	82	55	79
	Shared	80	41	60

DEMs2DEGs: DEGs targeted by DEMs. Bips_B: PBMC-derived hiPSCs vs. PBMCs, Fips_F: SF-derived hiPSCs vs. SFs, Uips_U: UC-derived hiPSCs vs. UCs.

## Data Availability

The command script for MirDeep2 and downstream analysis code written by R are available at the GitHub repository https://github.com/Andelyu/hiPSCs_exosomal_miRNA_project.
